# Comparing Biomechanical Properties of Bioabsorbable Suture Anchors: A Comprehensive Review

**DOI:** 10.3390/biomimetics10030175

**Published:** 2025-03-12

**Authors:** Dorien I. Schonebaum, Noelle Garbaccio, Maria J. Escobar-Domingo, Sasha Wood, Jade. E. Smith, Lacey Foster, Morvarid Mehdizadeh, Justin J. Cordero, Jose A. Foppiani, Umar Choudry, David L. Kaplan, Samuel J. Lin

**Affiliations:** 1 Department of Plastic and Reconstructive Surgery, Harvard Medical School, Beth Israel Deaconess Medical Center, Boston, MA 02215, USA; dschoneb@bidmc.harvard.edu (D.I.S.); ngarbacc@bidmc.harvard.edu (N.G.); mescoba2@bidmc.harvard.edu (M.J.E.-D.); sasha.wood@yale.edu (S.W.); jsmith70@bidmc.harvard.edu (J.E.S.); lfoster4@bidmc.harvard.edu (L.F.); mmehdizadeh@hms.harvard.edu (M.M.); foppi002@umn.edu (J.A.F.); 2 Department of Plastic and Reconstructive Surgery, Amsterdam UMC, 1105 AZ Amsterdam, The Netherlands; 3 Department of Plastic and Reconstructive Surgery, University of Minnesota, Minneapolis, MN 55455, USA; choud008@umn.edu; 4 Department of Biomedical Engineering, Tufts University, Medford, MA 02155, USA; david.kaplan@tufts.edu

**Keywords:** suture anchor, absorbable suture, non-absorbable suture, all-soft, all-suture, silk, orthopedic surgery, biocompatibility, strength, soft tissue, osteolysis, cyst, rotator cuff repair, arthroscopic

## Abstract

Suture anchors (SAs) are medical devices used to connect soft tissue to bone. Traditionally these were made of metal; however, in the past few decades, bio-absorbable suture anchors have been created to overcome revision surgeries and other complications caused by metallic SAs. This systematic review aims to analyze the biomechanical properties of these SAs to gain a better understanding of their safety and utilization. A comprehensive systematic review that adhered to the PRISMA guidelines was conducted. Primary outcomes were that the pull-out strength of SAs, the rate of degradation secondarily, and the biocompatibility of all SAs were analyzed. After screening 347 articles, 16 were included in this review. These studies revealed that the pull-out strength of bio-absorbable SAs was not inferior to that of their non-absorbable comparatives. The studies also revealed that the rate of degradation varies widely from 7 to 90 months. It also showed that not all absorbable SAs were fully absorbed within the expected timeframe. This systematic review demonstrates that existing suture anchor materials exhibit comparable pull-out strengths, material-specific degradation rates, and variable biocompatibility. All-suture anchors had promising results in biocompatibility, but evidence fails to identify a single most favorable material. Higher-powered studies that incorporate tissue-specific characteristics, such as rotator cuff tear size, are warranted. To meet demonstrated shortcomings in strength and biocompatibility, we propose silk fibroin as a novel material for suture anchor design for its customizable properties and superior strength.

## 1. Introduction

Suture anchors (SAs) are small surgical devices that are often utilized in the fields of plastic and orthopedic surgery [1]. SAs are tools that allow the secure fixation of soft tissues, such as tendons and ligaments, to bone [2]. Rotator cuff repairs, ACL repairs, and Bankart repairs are among the many procedures that frequently utilize SAs [3,4]. Historically, SAs were made from metals such as titanium or stainless steel. Metals proved to be strong and rigid, but long-term disadvantages were realized. Over time, metallic SAs can migrate or loosen, damaging cartilage as they move through the joint. Their non-absorbable nature lends to artifacts that obscure imaging results, and their persistence complicates revision surgery, challenging surgeons to avoid or remove anchors that are protected by scar tissue. These shortcomings prompted the development of other non-absorbable SAs and bioabsorbable suture anchors [3].

Two commonly used types of non-absorbable suture anchors are the all-soft suture anchors (ASAs), and polyetherketone (PEEK) SAs. ASAs are made of ultra-high-molecular-weight polyethylene and were designed to reduce complications associated with metal and other non-absorbable suture anchors. Tightening the first suture of an ASA self-tethers the anchor within the osseous cavity, locking the ASA in place. The primary disadvantage of an ASA is weaker fixation, leading to more laxity in the joint [5]. PEEK SAs are designed from a synthetic thermoplastic polymer [6]. Their pull-out strength is measured to be similar to that of metal, and their radiolucency avoids artifacts on imaging. They are preferable to metallic SAs as they are radiolucent; however they are not made of metal and therefore have a decreased chance of damaging tissue when they do start to migrate [7].

In the 1970s, the first SA constructed out of biomaterials was developed. Materials used for these early SAs were poly-lactic acid (PLA) and polyglycolic acid (PGA). PGA was the first biomaterial used, but its tendency to degrade within one week of placement led to quick discontinuation. The rapid degradation profile weakened fixation strength and increased the risk of inflammation secondary to PGA particles separating from the anchor [5]. PLA anchors were designed to replace PGA anchors. PLA is a material that takes up to five years to fully degrade. PLA SAs successfully improved upon PGA’s long-term strength, but the slow rate of degradation introduced new complications; the slow degradation compromised the ability for full bone replacement, ultimately limiting the strength of fixation after full degradation [5].

β-tricalcium phosphate (bTCP) is often used as a byproduct in absorbable SAs for its osteoconductive properties. Too weak to be used alone, bTCP serves to increase bone tissue growth when integrated with other SA materials in small percentages. The introduction of bTCP has enhanced the long-term bone regeneration of SA sites, preparing the bone to maintain fixation strength even after the SA degrades [8].

The improvements and ongoing challenges witnessed in SA design highlight the most important properties of an effective suture anchor. The ideal SA facilitates strong mechanical fixation, degrades fully when no longer needed, integrates into the bone, and minimizes inflammatory response [5,9]. The aim of this comprehensive review is to gain an understanding of the current literature describing the mechanical properties of absorbable SAs and comparing different types of SAs. This comparison will allow for a unified understanding of the current state of SA design, existing weaknesses, and future directions for optimal surgical outcomes. A literature review will be performed to analyze the use of bio-absorbable suture anchors in the medical fields of plastic and orthopedic surgery.

## 2. Materials and Methods

### 2.1. Systematic Review

#### 2.1.1. Information Sources

A detailed systematic literature review was performed using articles from Medline, Cochrane Central Register, Embase, and Web of Science. The review was performed in November and December 2024. The Preferred Reporting Items for Systematic Reviews and Meta-Analysis (PRISMA) guidelines were followed [10].

#### 2.1.2. Search Strategy

The search strategy was designed with keywords to include all studies with bioabsorbable suture anchors. Search terms included, but were not limited to, “suture anchor”, “bioabsorbable”, “biodegradable”, “biomaterial”, “PLA”, and “PGA”. The full search strategy can be found in Table 1. The search strategy was created by the researchers and thoroughly checked by a professional librarian who uploaded all articles into Covidence, a systematic review software.

#### 2.1.3. Eligibility Criteria

Observational studies and clinical trials were eligible for inclusion if they reported original, primary data on the biomechanical properties of bioabsorbable suture anchors. Eligible articles had to include a human (cadaveric or in vivo) adult population and report on the biomechanical properties of bioabsorbable suture anchors. Eligible languages were limited to English, Spanish, and Dutch. Exclusion criteria included non-human studies, editorials, letters to the editor, and reviews without primary data. The full detailed version of the eligibility criteria can be found at PROSPERO under the registration identification number CRD42024578743 [11].

#### 2.1.4. Selection Process

The results of the database query were uploaded to Covidence, an online systematic review program [12]. Three independent reviewers (N.G., D.S., and S.W) performed a two-stage screening process. The first stage consisted of title and abstract screening. In the second stage, the same reviewers performed a full text analysis of the remaining articles. A third reviewer (N.G., M.E.) resolved any discordances that occurred.

#### 2.1.5. Data Extraction

An extraction table in Microsoft Excel was created for data extraction. Three independent reviewers (N.G., D.S., and S.W.) extracted the data from all selected articles. Variables included the first author’s last name, country, publication year, study type, material of the suture anchor, type of surgery, strength of mechanical fixation, rate of degradation, imaging used to measure degradation, biocompatibility, and anything else that seemed noteworthy.

#### 2.1.6. Data Items

The primary outcome of this study was the determination of the main mechanical properties that contribute to effective SA design. These outcomes included mechanical fixation, the rate of degradation, and the regrowth of bone. Secondary outcomes focused on the biocompatibility of the suture anchors such as peri-anchor cyst formation and the osteolysis caused by the SA.

#### 2.1.7. Statistical Analysis

The outcome measures of the studies included in this systematic review varied widely. This eliminated the possibility to do any further statistics beyond descriptive ones.

#### 2.1.8. NIH Quality Assessment

The National Institute of Health has created a quality assessment tool to evaluate potential for bias in the included studies, which will be ranked as “poor”, “fair”, or “good” [13]. The quality assessment can be found in Table 2.

### 2.2. Literature Review

#### 2.2.1. Search Strategy

A comprehensive literature review was performed using keywords, subject headings, and controlled vocabulary to search Medline/PubMed and Web of Science including studies published until November 2024.

#### 2.2.2. Study Selection

Relevant studies describing possible usages for bio-absorbable SAs were selected as part of the literature review performed.

#### 2.2.3. Data Extraction/Synthesis

A narrative analysis of the included study was performed to serve as a framework to understand the usage of bio-absorbable suture anchors in the fields of plastic and orthopedic surgery.

## 3. Results

### 3.1. Systematic Review

#### 3.1.1. Study Selection

A total of 347 records were identified. After removing duplicates, 308 abstracts and titles were screened. Of these, 69 full-text articles were sought for retrieval and assessed for eligibility. Of these, 53 studies did not meet inclusion criteria and were excluded. Ultimately, 16 studies were selected for inclusion and analyzed (Table 3). 

**Figure 1:**
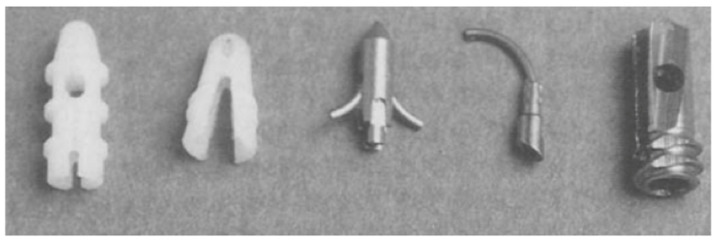
(From left to right) Acufex Rod TAG, Acufex Wedge TAG, Mitek GII, Mitek GI, and Statak.

A detailed overview of the sutures anchors can be found in Figure 1 [14].

#### 3.1.2. Study Characteristics

Included articles were published from 1998 to 2020. Most studies were conducted in Europe (56.2%), followed by Asia (25%), and the United States (18.8%). The main experimental approaches employed were in vivo (68.7%), followed by ex vivo approaches (12.5%), and case series (12.5%). Additionally, most studies assessed outcomes related to rotator cuff repair (56.2%) and open Bankart reconstruction (25%). A detailed overview of the general characteristics of the included studies can be found in Table 4.

#### 3.1.3. Suture Anchor Properties: Types of Anchors Used

Of the 16 included studies, most designs utilized SAs composed of a combination of biomaterials. The most commonly used biomaterials were Poly-L-lactic acid (PLLA), PLA, PGA, bTCP, Poly-L-lactide-co-D-L-Lactide (PLDLLA), calcium sulfate, and Poly-lacticglycolic acid (PLGA). At least one of these biomaterials was found in the SA design of each included study. Seven studies compared bioabsorbable and non-absorbable SAs. The most commonly used materials for non-absorbable SAs were PEEK, titanium, ASAs, and polyacetal. An overview of specific SAs used in each study can be found in Table 3.

#### 3.1.4. Strength

It was found that 5 of the 16 studies commented on the maximum pull-out strength of the SA. Pietschmann et al. 2008 reported an average ultimate failure load of 150 N for both the PLLA and the metal suture anchors; they reported no significant difference between the different anchors [15,16]. In 2009, the same group published another study reporting an ultimate failure load of 274 N for PLLA, 192 N for PLA, and 188 N for titanium SAs for non-osteopenic bones. The pull-out strength for PLLA was significantly higher than that of PLA and titanium SAs. The authors also performed the tests on osteopenic bones, which showed a significantly lower pull-out strength of 171 N for PLLA SAs, indicating that the SPIRALOK SA is safer to use on non-osteopenic bones [17,18,19,20,21,22]. Li et al. described an ultimate failure load of the PLLA SA of 365.83 N and a yield load of 281.83 N which was significantly higher than the PLLA screw comparison they used [23]. Haneveld et al. reported a similar pull-out strength for both PLLA and PEEK Sas; however, no specific values were mentioned [19]. Chen et al. did not mention specific values but described the pull-out strength of their PLDLLA SA to be greater than that of a PLLA SA produced by the same manufacturer [24] (Table 5).

#### 3.1.5. Degradation

Eleven out of the sixteen articles reported results on degradation. Degradation was assessed via MRI in six studies, via radiographs in four studies, and via MRI and CT in one study. Lee et al. and Haneveld et al. both noted no degradation of PEEK SAs [18,19]. Lee et al. compared PEEK SAs to biocomposite anchors, neither of which degraded within the 6-month follow up period [18]. Di Benedetto et al. also tested biocomposite PGA/bTCP SAs; after 12 months, 82% of the biocomposite SAs were barely visible on MRI [20]. Other studies that investigated PLLA/PGA/bTCP SAs observed a degradation of 68% resorption at 12 months [25], 73.7% resorption at 18 months [21], 98% resorption at 24 months [25], and 34% resorption at 28 months [19]. Vonhoegen et al. studied PLGA/bTCP/calcium sulfate SAs and reported 50% of the SAs to be fully reabsorbed within 21 months [26]. Ejerhed et al. observed 44% of PGA/bTCP SAs to be fully absorbed at 7 months post-op. This increased to 56% at 33 months; the remaining 44% showed visible or cystic drill holes at 33 months, although the visibility had decreased over time [27]. Sgroi et al. investigated 85% PLLA + 15% bTCP SAs, 90% of which persisted on imaging after 2 years; however, the authors note that the SAs were designed for slow degradation and osseous integration, both of which were seen on the MRI scans [17]. Warme et al. compared PGA/PLA copolymer SAs to polyacetal non-absorbable SAs. Radiographs showed that the PGA/PLA copolymer was completely absorbed after 1 year, whereas the non-absorbable SAs were fully present [15]. Magnusson et al. followed outcomes in PGA/bTCP SAs for the longest follow-up period of all studies at 90 months post-op. Radiographs showed that 66% PGA/bTCP SAs were fully absorbed at 90 months, and 34% of SAs remained visible or cystic [28]. Kartus et al. compared the effect of surgical technique, open vs. arthroscopic, on the rate of degradation. At 24 months post-op, the open surgery group showed significantly more visible drill holes than the arthroscopic group. Of all SAs, 27.8% had fully been absorbed, 33.3% were classified as hardly visible on radiographs, 13.9% had visible drill holes, and 25% showed visible drill holes with cystic formation [29] (Table 6).

#### 3.1.6. Biocompatibility

A total of 13 studies commented on biocompatibility. Overall, all SAs studied in these articles demonstrated some extent of biocompatibility mostly in the form of complication description. The most commonly observed complications were osteolysis and peri-anchor cyst formations. Warme et al. observed an initial increase in drill hole size at 6 months post-op that disappeared by 12 months post-op [15]. Single-case findings of osteolysis were reported in PLLA by Sgroi et al., and in PEEK by Haneveld et al. [17,19]. Di Benedetto et al. reported that 12% of all cases showed minimal signs of osteolysis [20], and Vonhoegen et al. reported that 2.4% of cases showed small osteolytic structures [26]. Another four studies reported on peri-anchor cyst formation. Lee et al. found cyst formation in 72.4% of biocomposite SAs and 58.6% of PEEK SAs at 3 months post-operatively. At 6 months, these findings had increased to 75.9% and 72.4%, respectively. They concluded that cyst formation occurs significantly faster with biocomposite SAs, this rate however stabilizes at 6 months [18]. Chung et al. also reported peri-anchor cyst formation in 60% of cases at 6 months after placement; cyst formation significantly decreased to 18.4% at 18 months [21]. Milewski et al. observed cyst formation in 6.3% of cases after 24 months [25]. Magnusson et al. reported that 33% of cystic drill holes persisted at 90 months [28]. Two more studies commented on peri-anchor fluid formation. Kim et al. reported the highest rate of peri-anchor fluid formation in the PEEK SA group, which they described as unexpected [30]. Kartus et al. and Magnusson et al. reported on the degeneration of the joint. Kartus et al. investigated open versus arthroscopic uses of SAs, and they observed that open surgery was associated with more signs of degeneration at 24 months post-operation [29]. Magnusson et al. documented an increase in degenerative changes at 7, 33, and 90 months post-operation [28]. The studies of Chen et al. and Ejerhed et al. were the only two studies that reported surgical complications with one case of implant failure, five cases of skin infection, one wound infection, and one incident of DVT [24,27]. All other studies commenting on the success of the SA observed no mispositioning or migration [17,19,20]. 

### 3.2. Literature Review

#### 3.2.1. First-Generation Suture Anchors

The first suture anchor was No. 2 braided polyester bonded to a headless titanium hex screw. Goble and Somers patented and marketed the device as Statak (formerly Zimmer, Warsaw, IN, USA) in 1985. Statak was designed to take advantage of the strength of a threaded screw and the accessibility of a suture for Bankart labral repair in the shoulder [31]. From 1985 to 1992, five suture anchors became available on the market: Statak, Mitek G1, Axufex Rod, Acufex Wedge, and Mitek G2 [14].

Most first-generation SAs utilized metallic materials. The Mitek (formerly Mitek Inc., Canton, OH, USA) SA relied on the properties of nitinol (nickel-titanium alloy). The nitinol allowed the Mitek’s curved structure to linearize when entering a predrilled hole; in cancellous bone, the Mitek returned to a natural arc, “locking” on to the edge of the cortex [32]. The 1987, Mitek G1 had one nitinol arc; it was succeeded in 1992 by Mitek G2, which featured double nitinol arcs to improve pull-out strength. The Mitek SAs were the first SAs formally investigated. Richmond et al.,reported the results of 32 patients who underwent a modified Bankart reconstruction with Mitek G1 anchors, finding no complications and a success rate that was comparable to that of traditional Bankart repair performed with suture tunnels [32]. The four participating surgeons reported that SAs simplified the procedure, lending credence to the earliest iterations of SAs.

In the same year as the first SA publication, the Acufex Rod and Wedge (Acufex Microsurgical Inc., Norwood, MA, USA) became available [33]. Made of polyacetal (non-absorbable plastic), the Acufex Rod and Wedge were part of the Tissue Anchor Guide (TAG) system. “Rod” and “wedge” describe the implant shapes, both designed with a diameter wider than that of the predrilled hole but fit for separate surgical indications. The non-absorbable plastic was the favored material in patients with metal allergies.

#### 3.2.2. Biomechanics of Suture Anchors

The results of the first in vivo biomechanical tests comparing first-generation SAs were published in 1993 [14]. Carpenter et al. tested the devices to failure in human cadaveric tibiae for a total of 198 trials. Pull-out strengths were measured as the mean load at which the anchor pulled out of bone. At a diameter of 0.5 mm, Mitek G1 was the smallest of the cohort and had the lowest pull-out strength of 49.4 N. The TAG Rod and Wedge had intermediate pull-out strengths of 67.2 and 65.5 N. Statak and Mitek G2 exhibited the highest pull-out strengths of 90.2 N and 82.4 N, respectively. These early prototypes mostly failed when the suture broke, as the pull-out loads were greater than the strength of the suture (commonly No. 2 Ethibond, which has a strength of ~90 N) [34].

The biomechanics of SAs have since come to be understood as an interplay of SA size and the suture chain. Smaller anchor sizes, and corresponding reductions in drill diameter, are correlated to decreased pull-out strengths [35]. The suture chain consists of connections from anchor to bone and suture to soft tissue. To prevent anchor pull-out, the anchor-to-bone fixation should be the strongest link in the suture chain, meaning that the anchor supports a higher load than the tensile strength of the suture. To prevent suture breakage, the suture should then be stronger than the soft tissue [36]. Prioritizing anchor and suture strength in SA design ensures that SA failure occurs at the tissue level, which is less destructive to the bone than other modes of anchor failure and may still allow for healing or partial function.

To increase suture strength, manufacturers began to load SAs with multiple sutures, but braided polyester was still limited to failure loads of ~90 N. Ultrahigh molecular weight polyethylene (UHMWP) sutures were introduced in the early 2000s, doubling the failure strength to ~180 N [37]. In response to the stronger suture strength, screw designs were re-imagined to strengthen anchor-to-bone fixation. Threaded screw designs, expanding anchors, and interference fixation techniques upgraded the failure loads of metallic SA to be as high as 800 N, measured in the AME 5.5 (1995; AME International, Vienna, Austria) [37].

#### 3.2.3. Biodegradable and Biocomposite Materials

Anchor migration, interference with diagnostic imaging, and suitability for pediatric patients presented as early concerns for the use of metallic SAs, ushering in the development of bioabsorbable SAs [38]. The first biodegradable SA were made of polyglycolic acid (PGA), poly-lactic acid (PDLA/PLLA), or copolymers of PGA and PLLA (PLGA). Relatively high incidences of inflammatory reactions and cyst formation were identified in the most rapidly degrading materials, namely PGA and PGLA. PLLA is the favored biodegradable material for its longer degradation time of more than two years [39]. Bioabsorbable SAs were structurally weaker than metal SAs, and they were more likely to fail by eyelet breakage. Studies debated the ability of biodegradable materials to withstand physiological forces in vivo, but the clinical outcomes of biodegradable and metallic SAs have been comparable [40,41,42].

Non-degradable plastic polyetheretherketone (PEEK) SAs were also popularized in the 2000s to address the weaker fixation strength of biodegradable SAs. Like biodegradable materials, PEEK did not interfere with imaging, yet its enhanced range of fixation strength from 168 N to 605 N was reminiscent of metallic SAs. With robust mechanical properties and high strength, PEEK is a commonly employed material in contemporary surgery [43]. PEEK is primarily limited by its poor osseointegration.

Instead of being replaced by bone, biodegradable materials are reconstituted as calcified fibrous tissue, and PEEK is not absorbed at all [44]. Biocomposite SAs were developed to improve tissue integration, incorporating calcium phosphates, like β-tricalcium phosphate (β-TCP) and hydroxyapatite (HA), to promote bone ingrowth. Structurally derived from natural bone mineral content, calcium phosphates are highly biocompatible with human bone [45]. Accordingly, biocomposite SAs have fast degradation rates but successfully facilitate bony ingrowth, identified as bony bridging and trabecular formation in long-term imaging [46].

#### 3.2.4. Evolution of SA Appearance

The shape and size of different suture anchors has evolved over time, from screw-like titanium structures in the first Statak anchors to different creative designs manufactured by Mitek. Figure 1 shows an overview of these SAs. After experimenting with different shapes, mostly those made by Mitek, a general consensus was reached. Most SAs are slightly larger than the pre-drilled holes they are placed in for a secure lock. Their shape resembles the shape of a screw with locking features on each side to maximize pull-out strength by improving the grip of the SA on the bone [2,14].

#### 3.2.5. Suture Anchors in Specific Surgical Applications

The 1990s illustrated the fixation strength of suture anchors was non-inferior to that of transosseous tunnels and staples in numerous surgeries, starting with Bankart lesions and rotator cuff repairs [31,47]. In 1991, Wolf et al. described arthroscopic capsulolabral repair using suture anchors, offering a simpler and quicker surgical technique than traditional Bankart repair [48]. In 1996, Craft et al. and Reed et al. compared the strength of Statak and Mitek superanchors (incorporating four prongs; Mitek Inc.) to transosseous tunnels in rotator cuff repair; the SA repairs were significantly stronger than the standard suture technique.

Arthroscopic surgery with SAs was quickly appreciated for its reduced surgical complexity, enhanced patient comfort, and improved post-operative outcomes. Manufacturers adapted SA lengths to expand the breadth of application and reduce iatrogenic damage in more delicate anatomy. SA applications were documented in the shoulder to the wrist, hand, and foot before the start of the 21st century [49,50]. The MiniMitek (Mitek Inc.), released in 1995, was among the first of “mini” suture anchors, defined as anchors that create a bone defect of an internal diameter that is less than 2.2 mm [51]. Given that it is 5.4 mm in length and 1.8 mm in diameter, the MiniMitek broadened marketed applications to include midfoot/hallux reconstruction, thumb collateral ligament reconstruction, and scapholunate reconstruction [52]. Similarly, the MicroMitek (Mitek Inc.) was developed in 1996 for hand reconstruction, made possible by its short 3.7 mm length.

#### 3.2.6. Innovations and Future Directions

Novel designs in SAs aim to maximize pull-out strength, optimize osteoconductive properties, and enable time degradation to occur slowly enough to stabilize the tissue during the physiological healing process and minimize inflammation [53,54]. Modern SAs utilize lessons learned from previous designs by incorporating a mix of materials. For example, Vonhoegen et al. described a novel biocomposite suture anchor for rotator cuff repair in 2019 that consists of 65% PLGA, 15% β-TCP, and 20% calcium sulfate (CS) [54]. PLGA maintains stability and proffers nearly complete SA absorption within 24 months, while bioceramics promote bone-laying and degrade quickly to minimize inflammation. Of 82 PLGA/β-TCP/CS SAs placed in 48 patients, Vonhoegen et al. reported zero incidents of severe osteolysis or cyst formation, zero anchor pull-out complications, and only two soft tissue re-tears [54].

Knotless SAs and all-suture anchors are additional SA innovations developed to simplify arthroscopic surgery and reduce SA-related risk of arthritis, respectively. Arthroscopic knot-tying presented a steep learning curve in the operating room. In 2001, Thal proposed the first knotless SA, the Mitek Knotless (Mitek Inc.) [54]. The Mitek Knotless had a titanium body; later iterations of knotless SAs adopted biodegradable and biocomposite materials.

Knotless SAs eliminated the need for knot-tying, making arthroscopic orthopedic surgeries more accessible and reducing operative time. Knotless SAs did not address the pro-arthritic potential of SAs, which is especially prevalent in metallic SAs. All-suture anchors, or all-soft SAs, were designed to be small, soft, and knotless. The design preserved bone and tissue integrity and eliminated knots that may impose local pressure, theoretically decreasing arthritic potential. Limited evidence on all-suture anchors points to promising biocompatibility, and early biomechanical testing suggests pull-out strengths that are comparable to those of solid anchors [55]. Of our studies, only Kim et al. compares in vivo outcomes of all-suture anchors and PEEK anchors, finding less peri-anchor fluid collection and osseous inflammation in all-suture anchors than in PEEK anchors.

Future directions focus on developing new and enhanced SA materials to optimize suture chain mechanics and biocompatibility. The SA landscape is still absent an SA design that balances pull-out strength, resorption time, tissue preservation, and favorable long-term clinical outcomes.

## 4. Discussion

This study reflects on the decades of effort expended to optimize suture anchor strength and biocompatibility to reduce post-operative complications. Since their introduction in the 1970s, suture anchors have undergone remarkable evolutions to meet the demands of soft tissue fixation to bone in both open and arthroscopic surgery [5]. Functioning as a secure point of fixation—tethering soft tissue to bone—suture anchors are the gold-standard technique in many orthopedic surgeries. Irrespective of surgical and anatomic application, the most effective suture anchor design maintains two primary goals: (1) to maximize pull-out strength and (2) to minimize acute and chronic tissue inflammation. Mediating strength and biocompatibility, optimal degradation timing is a secondary goal; suture anchors that degrade too quickly can compromise strength, yet degrading too slowly can compromise biocompatibility.

The earliest suture anchors were predominantly non-absorbable, utilizing materials like stainless steel and titanium. While these exhibited high mechanical strength and consistent long-term performance, they could not contribute favorably to tissue healing and bone integration. Long-term complications related to implant migration and poor tissue compatibility prompted the development of absorbable anchors. Absorbable designs incorporated PGA, PLLA, and other materials that improved biocompatibility without sacrificing mechanical strength. Introduced in the 2000s, biocomposite and all-suture anchors are more contemporary options. Biocomposite anchors take advantage of osteoconductive minerals like calcium sulfate to minimize cystic reactions and facilitate bone integration. All-suture anchors, although non-absorbable, are composed entirely of sutures and feature high biocompatibility with minimal inflammation.

Our results demonstrate that the pull-out strength is comparable across metal, PLA, and PLLA suture anchors. Only one study found that PLLA was significantly favorable to PLA and titanium in non-osteopenic bone, but differences were not significant in osteopenic bone [22]. While it is tempting to infer tha bioabsorbable PLLA is non-inferior and even superior to non-absorbable suture anchors in non-osteopenic bone, the results are weakened by a small sample size of twelve cadavers and moreover not supported by other comparisons of these materials.

The degradation profiles of suture anchors depended strongly on their material design. Fast-degrading materials, like PLA/PGA or PLLA/PGA with β-TCP, are expected to substantially absorb within 12–24 months of installation. In defining “substantial” absorption as at least a 50% decrease in visible suture anchors on MRI or radiograph at 12–24 months post-op, 8 of 11 studies investigating degradation agreed with the proposed time frame [15,18,20,21,25,26,28,29]. However, this is a liberal application of “substantial” absorption, and true rates will vary by tissue type and material. PLLA-based anchors are designed to degrade more slowly to prolong mechanical stability; indeed, Haneveld et al. and Sgroi et al. demonstrated that PLLA-predominant sutures mostly maintained their structures after two years [17,19]. However, like non-absorbable suture anchors, the PLLA-based anchors have persistent drill holes that produce artifacts on imaging, and their associated fluid formation implicates an inflammatory response. These complications challenge the benefits of slow degradation. Ideally, rates of degradation should align with the bone healing process, allowing suture anchors to (1) give stability until the bone is healed then (2) integrate into tissue to prevent long-term inflammation and imaging artifacts. Suture anchors are engineered with bone healing time in mind, but the healing process is tissue- and trauma-specific, precluding a one-size-fits-all design.

The suture anchors studied were biocompatible to the extent that complications rarely carried functional consequences. Peri-anchor cyst formation was the most common complication observed. Cyst formation was more prevalent in biocomposite anchors than in non-absorbable designs, like those made composed of PEEK. Rates of cyst formation were highest within the first 6 months of repair and declined with time; most cysts regressed by 18–24 months post-operatively and did not correlate with degenerative changes or adverse clinical outcomes. Peri-anchor fluid collection was also observed in both absorbable and non-absorbable anchors, although studies disagreed on whether fluid formation was more prevalent in PLLA or PEEK anchors [19,30]. Only Kim et al. investigated the biocompatibility of all-suture anchors, finding less peri-anchor fluid collection and osseous inflammation in all-suture anchors than in PEEK anchors, despite their shared non-absorbable nature [30]. Small osteolytic changes occurred in a minority of PEEK, PLLA + β-TCP, and biocomposite anchors, but they did not confer clinically meaningful changes in function or mobility [17,19,20,26,28]. The variable reports on peri-anchor cyst formation, fluid collection, and osteolytic changes confound any comparison of biocompatibility in non-absorbable and absorbable suture anchors. Notably, Chung et al. noted direct associations between cyst size and the retraction and anterior–posterior length of the rotator cuff tear, which may partially explain results documented in other studies [21]. The inconsistent reports of biocompatibility may also be influenced by differences in tissue type and procedure. All-suture anchors have promising biocompatibility, but the findings are limited to one study [30]. To better understand the interaction of soft tissue and SA biocompatibility, discrepancies warrant larger sample sizes, greater incorporation of all-suture anchors, and the inclusion of intraoperative findings, like rotator cuff tear size, in future investigations.

Our results reaffirm that the balance between strength and biocompatibility is still unrealized. Absorbable suture anchors were developed to overcome the shortcomings of early metallic suture anchors. While they do deliver similar strength, their degradation rates are not optimally adapted to the bone healing process, and their relative biocompatibility is inconsistent and ill-defined. All-suture anchors represent a newer non-absorbable design that may improve upon existing techniques, pending further investigations.

The limitations of this systematic review are numerous. The included studies evaluated many different surgeries, ranging from rotator cuff repair to the arthroscopic fixation of tibial eminence fractures. Some study designs involved cadavers, whereas those that followed clinical outcomes lacked homogeneity in patient age, surgery, and mechanisms of injury. Follow-up times also differed, limiting an objective understanding of the long-term clinical implications of early post-operative osteolytic changes, cyst formations, and fluid collection. Studies were further complicated by small sample sizes and inconsistent approaches to imaging and strength testing. Consequently, the discussed results are not directly comparable, and their interpretations should be mindfully regarded.

To optimize suture anchor strength and biocompatibility, interested groups may consider pivoting to a novel material. Suitable SA candidates should exhibit fixation strength, biodegradability, and tissue compatibility that are comparable to those of existing SA materials. Advances in silk chemistry point to silk fibroin as a promising candidate [56,57]. Silk has already demonstrated successful applications in tissue engineering, drug delivery, cell coating, and biosensors, among other utilities. Importantly, the structure of silk can be modified through various bonding chemistries, enabling silk as a highly versatile material that can be fine-tuned to achieve specific mechanical and biochemical properties [56,57,58,59,60,61,62]. The strength-to-density ratio of silk is significantly greater than that of steel and Kevlar, currently the golden standard for fiber technology [4,10]. Investigating the pull-out strength, degradation, and biocompatibility of silk-based suture anchors would effectively explore the potential of this material in soft tissue fixation.

## 5. Conclusions

Suture anchors have evolved to incorporate different materials to balance strength, degradation, and biocompatibility for improved surgical outcomes. This systematic review demonstrates that existing materials exhibit comparable pull-out strength, material-specific degradation rates, and variable biocompatibility. Although the inherent nature of bone- and tissue-healing precludes the possibility of a one-size-fits-all suture anchor, the literature fails to identify a single most favorable material to date. Promising biocompatibility results in all-suture anchors warrant further exploration of all-suture anchors in high-powered studies that incorporate tissue-specific characteristics, such as rotator cuff tear size, which may be correlated with complications like peri-anchor cyst formation. Its customizable properties and superior strength position silk fibroin as a promising base to optimize suture anchor strength and biocompatibility in the future.

## Figures and Tables

**Table 1:** Search Strategy and Database Sources.

Database	Search
PubMed/ MEDLINE (AB = )	(((anchor, suture[MeSH Terms]) OR (anchors, suture[MeSH Terms]) OR (Suture Anchor[Title/Abstract]) OR (Anchor, Suture[Title/Abstract]) OR (Anchors, Suture[Title/Abstract]) OR (All soft suture anchor[Title/Abstract])) AND ((biomaterials[MeSH Terms]) OR (biomaterial[Title/Abstract]) OR (bioabsorbable[Title/Abstract]) OR (bioresorbable[Title/Abstract]) OR (poly-lactic acid[Title/Abstract]) OR (polyglycolic acid[Title/Abstract]) OR (ultra-high-molecular-weight polyethylene[Title/Abstract]) OR (biomaterials[Title/Abstract])))
Web of Science (AB = )	AB=(“suture anchor” OR “suture anchors” OR “anchor, suture” OR “anchors, suture” OR “all soft suture anchor”) AND AB=(“biomaterials” OR “biomaterial” OR “bioabsorbable” OR “bioresorbable” OR “poly-lactic acid” OR “polyglycolic acid” OR “ultra-high-molecular-weight polyethylene”)
Web of Science (TI = )	TI=(“suture anchor” OR “suture anchors” OR “anchor, suture” OR “anchors, suture” OR “all soft suture anchor”) AND TI=(“biomaterials” OR “biomaterial” OR “bioabsorbable” OR “bioresorbable” OR “poly-lactic acid” OR “polyglycolic acid” OR “ultra-high-molecular-weight polyethylene”)
Cochrane Central Register (AB+TI+AK = )	(“suture anchor” OR “suture anchors” OR “anchor, suture” OR “anchors, suture” OR “all soft suture anchor”) AND (“biomaterials” OR “biomaterial” OR “bioabsorbable” OR “bioresorbable” OR “poly-lactic acid” OR “polyglycolic acid” OR “ultra-high-molecular-weight polyethylene”)

**Table 2:** NIH Quality Assessment.

Article	Author	Score
Clinical and Radiologic Outcomes of Combined Use of Biocomposite and PEEK Suture Anchors During Arthroscopic Rotator Cuff Repair	J. Lee	Fair
Arthroscopic Double-Row Repair of the Rotator Cuff Using Bioabsorbable and Nonresorbable Anchors	H. Haneveld	Fair
Nonabsorbable Versus Absorbable Suture Anchors for Open Bankart Repair	W. Warme	Good
Biomechanical Testing of a Knotless Anchor Compared with Established Anchors for Rotator Cuff Repair	M. Pietschmann	Fair
Biocomposite Suture Anchors Remain Visible Two Years After Rotator Cuff Repair	M. Sgroi	Good
Reliability of Open Architecture Anchors in Biocomposite Material: Medium-Term Clinical and MRI Evaluation	P. Di Benedetto	Fair
Changes in Perianchor Cyst Formation Over Time After Rotator Cuff Repair	S. Chung	Good
Suture Anchor Fixation Strength in Osteopenic Versus Non-Osteopenic Bone for Rotator Cuff Repair	M. Pietschmann	Fair
Clinical Safety and Efficacy of a Novel Ultrasound-Assisted Bioabsorbable Suture Anchor in Foot and Ankle Surgeries	J. Chen	Good
Bone Replacement of Fast-Absorbing Biocomposite Anchors in Arthroscopic Shoulder Labral Repairs	M. Milewski	Fair
Arthroscopic and Open Shoulder Stabilization Using Absorbable Implants: A Clinical and Radiographic Comparison of Two Methods	J. Kartus	Fair
Absorbable Implants for Open Shoulder Stabilization: A 7–8 Year Clinical and Radiographic Follow-Up	L. Magnussen	Fair
Osteoconductive Resorption Characteristics of a Novel Biocomposite Suture Anchor Material in Rotator Cuff Repair	J. Vonhoegen	Good
Absorbable implants for open shoulder stabilization: A clinical and serial radiographic evaluation	L. Ejerhed	Fair
The Formation of Perianchor Fluid Associated With Various Suture Anchors Used in Rotator Cuff Repair	S. Kim	Good
Arthroscopic Fixation of Tibial Eminence Fractures: A Biomechanical Comparative Study of Screw, Suture, and Suture Anchor	J. Li	Good

**Table 3:** Suture Anchor Materials by Publication, Author, and Study Type.

Title of Publication	First Author, Country	Year	Study Type	Product
Nonabsorbable Versus Absorbable Suture Anchors for Open Bankart Repair	Winston, W.; USA	1999	*In Vivo*	Polyacetyl PGA/PLA copolymer
Biomechanical testing of a new knotless suture anchor compared with established anchors for rotator cuff repair	Pietschmann, M.; Germany	2008	*Ex Vivo*	UltraSorb: PLLA SuperRevo: Titanium BioKnotless: PLA
Biocomposite Suture Anchors Remain Visible Two Years After Rotator Cuff Repair	Sgroi, M.; Germany	2019	*In Vivo*	85% PLLA and 15% bTCP
Clinical and Radiologic Outcomes of Combined Use of Biocomposite and PEEK Suture Anchors during Arthroscopic Rotator Cuff Repair	Lee, J.; South Korea	2020	*In Vivo*	PEEK Biocomposite: PLLA/PGA 70% + bTCP 30%
Arthroscopic double-row repair of the rotator cuff: a comparison of bio-absorbable and non-resorbable anchors regarding osseous reaction	Haneveld, H.; Germany	2013	*In Vivo*	PLLA PEEK
Reliability of Open Architecture Anchors in Biocomposite Material: Medium-Term Clinical and MRI Evaluation	Di Benedetto, P.; Italy	2020	*In Vivo*	PEEK PGA/bTCP
Changes in Perianchor Cyst Formation Over Time After Rotator Cuff Repair: Influential Factors and Outcomes	Chung S.; South Korea	2018	*In Vivo*	Bio-composite (PLLA/PGA/bTCP)
Suture Anchor Fixation Strength in Osteopenic vs. Non-Osteopenic Bone for Rotator Cuff Repair	Pietschmann, M.; Germany	2009	*Ex Vivo*	SPIRALOK: PLLA UltraSorb: PLA SuperRevo: Titanium
Clinical Safety and Efficacy of a Novel Ultrasound-Assisted Bioabsorbable Suture Anchor in Foot and Ankle Surgeries	Chen, J.; USA	2020	*Case Series*	PLDLLA (Poly(L-lactide-co-D, L-lactide)
Bone Replacement of Fast-Absorbing Biocomposite Anchors in Arthroscopic Shoulder Labral Repairs	Milewski, M.; USA	2012	*Case Series*	PLGA 70%/bTCP 30%
Absorbable Implants for Open Shoulder Stabilization: A Clinical and Serial Radiographic Evaluation	Ejerhed, L.; Sweden	2000	*In Vivo*	TAG: PGA and bTCP
Arthroscopic and Open Shoulder Stabilization Using Absorbable Implants: A Clinical and Radiographic Comparison of Two Methods	Kartus, J.; Sweden	1998	*In Vivo*	TAG and Suratac: PGA bTCP
Absorbable Implants for Open Shoulder Stabilization: A 7–8-Year Clinical and Radiographic Follow-Up	Magnusson, L.; Sweden	2006	*In Vivo*	TAG: PGA and bTCP
Osteoconductive Resorption Characteristics of a Novel Biocomposite Suture Anchor Material in Rotator Cuff Repair	Vonhoegen, J.; Germany	2019	*In Vivo*	65% PLGA, 15% bTCP and 20% calcium sulfate
The Formation of Perianchor Fluid Associated With Various Suture Anchors Used in Rotator Cuff Repair	Kim, S.; South Korea	2019	*In Vivo*	Anchor A: 30% β-TCP + 70% PLGA Anchor B: All-suture Anchor C: 23% micro β-TCP + 77% PLA Anchor D: PEEK
Arthroscopic Fixation of Tibial Eminence Fractures: A Biomechanical Comparative Study of Screw, Suture, and Suture Anchor	Li, J.; China	2018	*In Vitro*	PLLA

**Table 4:** General Characteristics of Included Studies, Type of Surgery, and Follow-up.

	Total	%
**No. of Publications Included**	16	100
Year Range	1998–2020	
**Countries Publications Drawn From (%)**		
USA	3	18.8
Europe	9	56.2
Asia	4	25.0
**Experimental Approach (%)**		
In vivo	11	68.7
Ex vivo	2	12.5
In vitro	1	6.3
*Case series*	2	12.5
**Length of Follow-up** (months)		
Range, median	6–90	25
**Type of Surgery (%)**		
Rotator Cuff Repair	9	56.2
Open Bankart reconstruction	4	25.0
Other	3	18.8

**Table 5:** Maximum pull out strength of SA.

Article Title	1st Author; Year of Publication	Material of SA	Strength of SA
Biomechanical testing of a new knotless suture anchor compared with established anchors for rotator cuff repair	Pietschmann, M.; 2008	UltraSorb: PLLA SuperRevo: Titanium BioKnotless: PLA	Ultimate failure loads ranged from 90N (UltraSorb) to 225 N (BIOKNOTLESS RC and Super Revo) with an average of 150N; differences were not statistically significant
Arthroscopic double-row repair of the rotator cuff: a comparison of bio-absorbable and non-resorbable anchors regarding osseous reaction	Haneveld, H.; 2013	PLLA PEEK	No specific values mentioned but pull-out strength was similar for both materials
Suture Anchor Fixation Strength in Osteopenic vs. Non-Osteopenic Bone for Rotator Cuff Repair	Pietschmann, M.; 2009	SPIRALOK: PLLA UltraSorb: PLA SuperRevo: Titanium	Ultimate failure load non-osteopenic bone: SPIRALOK: 274 N (significantly higher); Super Revo: 188 N; UltraSorb: 192 N Osteopenic bone: SPIRALOK: 171 N; Super Revo: 150 N; UltraSorb: 151 N Significant difference in pullout strength for SPIRALOK based on bone density
Clinical Safety and Efficacy of a Novel Ultrasound-Assisted Bioabsorbable Suture Anchor in Foot and Ankle Surgeries	Chen, J.; 2020	PLDLLA (Poly(L-lactide-co-D, L-lactide)	No specific number but they mention that pull out strength was measured by the manufacturer and they compared it to their standard bio absorbable SA. The PLDLLA SA had a greater pull out strength.
Arthroscopic Fixation of Tibial Eminence Fractures: A Biomechanical Comparative Study of Screw, Suture, and Suture Anchor	Li, J.; 2018	PLLA	Ultimate failure load: Suture anchor: 365.83 N, Screw: 195.93 N; Yield load: Suture anchor: 281.83 N, Screw: 178.75 N

**Table 6:** Rate of degradation.

Article Title	1st Author; Year of Publication	Material of SA	Type of Imaging	Degradation
Nonabsorbable Versus Absorbable Suture Anchors for Open Bankart Repair	Warme, W.; 1999	Polyacetyl (non-absorbable) PGA/PLA copolymer	Radiographs	12 and 24 months: PLA/PGA copolymer SA’s fully resorbed Polyacetyl SA’s showed no sign of absorption.
Biocomposite Suture Anchors Remain Visible Two Years After Rotator Cuff Repair	Sgroi, M.; 2019	85% PLLA and 15% bTCP	MRI	90% of implants still visible after two years.
Clinical and Radiologic Outcomes of Combined Use of Biocomposite and PEEK Suture Anchors during Arthroscopic Rotator Cuff Repair	Lee, J.; 2020	PEEK Biocomposite: PLLA/PGA 70% + bTCP 30%	MRI	No resorption seen on PEEK and Biocomposite SA at 3 and 6 months post operatively
Arthroscopic double-row repair of the rotator cuff: a comparison of bio-absorbable and non-resorbable anchors regarding osseous reaction	Haneveld, H.; 2013	PLLA and PEEK	MRI	Average of 34% degradation after 28 months for the PLLA SA’s; PEEK maintained structure.
Reliability of Open Architecture Anchors in Biocomposite Material: Medium-Term Clinical and MRI Evaluation	Di Benedetto, P.; 2020	PEEK PGA/bTCP	MRI	PGA/bTCP SA’s: 12 months 18% of suture anchors still visible (Grade 2) 82% Barely visible, partially edematous bleaching. (Grade 3)
Changes in Perianchor Cyst Formation Over Time After Rotator Cuff Repair: Influential Factors and Outcomes	Chung S.; 2018	Bio-composite (PLLA/PGA/bTCP)	MRI	6 months: no resorption 18 months: 73.7% fully resorbed and 26.3% partially resorbed
Bone Replacement of Fast-Absorbing Biocomposite Anchors in Arthroscopic Shoulder Labral Repairs	Milewski, M.; 2012	PLGA 70%/bTCP 30%	MRI and CT	12 months: 68% resorption 24 months: 98% resorption
Absorbable Implants for Open Shoulder Stabilization: A Clinical and Serial Radiographic Evaluation	Ejerhed, L.; 2000	TAG: PGA and bTCP	Radiographs	7 months: 44% fully absorbed, 56% visible or cystic drill holes 33 months: 56% fully absorbed, 44% visible or cystic drill holes
Arthroscopic and Open Shoulder Stabilization Using Absorbable Implants: A Clinical and Radiographic Comparison of Two Methods	Kartus, J.; 1998	TAG and Suratac: PGA bTCP	Radiographs	Group A: Open surgery, Group B: Arthroscopy 24 months group A and B: 27.8% invisible drill holes 33.3% hardly visible drill holes 13.9% visible drill holes 25% visible drill holes with cystic formation Group A had significantly more visible drill holes at 24 months.
Absorbable Implants for Open Shoulder Stabilization: A 7–8-Year Clinical and Radiographic Follow-Up	Magnusson, L.; 2006	TAG: PGA and bTCP	Radiographs	7-month: 11% invisible/hardly visible 89% visible/cystic 90-month: 66% invisible/hardly visible 34% visible/cystic Degradation occurred more slowly than in-vivo material testing of 25% degradation/week
Osteoconductive Resorption Characteristics of a Novel Biocomposite Suture Anchor Material in Rotator Cuff Repair	Vonhoegen, J.; 2019	65% PLGA, 15% bTCP and 20% calcium sulfate	MRI	21 months: 50% fully absorbed
